# The Role of EFL Teachers' Optimism and Commitment in Their Work Engagement: A Theoretical Review

**DOI:** 10.3389/fpsyg.2021.830402

**Published:** 2022-02-03

**Authors:** Yan Dong, Jieping Xu

**Affiliations:** School of Foreign Languages, Chaohu University, Chaohu, China

**Keywords:** English teaching, teacher optimism, teacher commitment, work engagement, positive psychology

## Abstract

Teachers' emotional states such as optimism and commitment have long been approved influential in second/foreign language education. Although many correlational investigations have been conducted on teacher optimism and commitment, their interaction and kinship with teachers' work engagement have been largely ignored in the literature. Considering this situation, the present mini-review aims to present the theoretical underpinnings, definitions, dimensions, and conceptualizations of these three important variables taken from positive psychology. Moreover, the present review can offer a number of practical implications for EFL teachers, teacher educators, program designers, school principals, and L2 researchers, and raise their awareness of the impact of inner emotions on teachers' academic performance. Finally, research gaps and future directions are provided for eager researchers to run similar and complementary studies in EFL contexts.

## Introduction

Undoubtedly, teaching is one of the most demanding and emotionally-tense occupations in the world whose success depends on numerous internal and external factors (McIntyre et al., 2017; Benevene et al., 2020). Effective instruction is, hence, the result of an interplay of the group and psychological variables. The complexity and tension in second/foreign language teaching double as teachers need to grapple with many cultural and psycho-emotional issues aside from linguistic disparities. Nevertheless, there are still many EFL teachers who work passionately, devotedly, and zealously to work their way out and generate positive outcomes in education. This steadiness and perseverance in EFL teachers boomed with the arrival of positive psychology (PP) which highlighted human's positive sentiments, agencies, properties, and conditions by which one can incredibly flourish and prosper (Seligman, 2011; MacIntyre et al., 2019; Wang et al., 2021). This trend in educational psychology rightly pointed to the power of positive emotions/factors in leading to academic success. It called for teachers to maintain a balance between their teaching ability and awareness of psychological factors influencing their instruction so that they can survive in the face of multiple adversities.

A classroom culture oriented to positive emotions and characteristics tackles weakness and establishes a sense of optimism in teachers by which they can execute instruction efficiently. Optimism is one of the most significant constructs proposed by PP includes hope, obligation, and a positive attitude toward life and career (Seligman, 2006). It is critical in language education in that an optimistic teacher looks on the bright side of his/her profession, looks for solutions to the existing problems, and maneuvers over the strengths and positive features of students, classes, schools, and networks (Pajares, 2001; Pathak and Lata, 2018). Optimism, in academia, is a collective sense of beliefs about the strengths and positive points of a school and its constituent elements, namely teachers, students, staff, facilities, connections, and the like (Safari and Soleimani, 2019). A teacher armored with such features sees opportunities in difficulties and takes constructive steps to transmit knowledge to his/her students. Research shows that optimism causes various positive outcomes in L2 education including teachers' and students' improved confidence, self-efficacy, classroom rapport, resilience, well-being, involvement, and academic performance/success (Smith and Hoy, 2007; Hoy et al., 2008; Hoy and Tarter, 2011; Sezgin and Erdogan, 2015; Lu, 2021).

Another offshoot of teacher optimism is a sense of academic commitment which is the most basic element of effective teaching (Lu, 2021). It refers to the extent to which teachers feel happy with their work and push themselves to indicate improved job execution (Altun, 2017). In other words, commitment is a core power related to teachers' work performance, attendance, continuation, and inclination toward success and accomplishments (Crosswell and Elliott, 2004). This construct is affected by both internal (personal) and external (contextual) factors that influence a teacher's work execution and teaching quality (Huang et al., 2016). A committed teacher shows zeal toward taking additional responsibilities and accepts the objectives of the school/university that he/she is working in with passion and energy (Sarikaya and Erdogan, 2016). This strong sense of obligation can help EFL teachers pass through the adversities and tensions common in L2 education. Moreover, commitment together with optimism can increase teachers' work engagement as a new concept proposed and scrutinized by PP adherents. The concept of work engagement pertains to job satisfaction, concentration, productivity, positive aspiration, resilience, and adaptability (Greenier et al., 2021). It is the opposite image of burnout that reflects teachers' professional career's quality (Minghui et al., 2018). Teachers' work engagement as a positive mental state has been found to affect their self-efficacy, reflectivity, self-regulation, and well-being (Burić and Macuka, 2018; Greenier et al., 2021; Han and Wang, 2021). Like other PP variables, it is affected by both emotional and professional factors. This led to numerous studies on the construct the majority of which are correlation. What seems to be missing, in the literature, is exploring the interactional and dynamic interplay of teachers' optimism, commitment, and work engagement. Urged by this gap, this review study aimed to present the theoretical background of each variable and their possible association and interaction in EFL contexts.

## Background

### The Concept of Optimism

The notion of optimism has its roots in Bandura's social intellectual hypothesis, Coleman's community principal hypothesis, and Seligman's academic optimism. It is a novel construct introduced in PP research that strongly and directly influences teachers' pedagogy and students' achievement. Optimism comprises collective efficacy, faculty trust, and academic emphasis (Asgari and Rahimi, 2014). It is an intrinsic attribute referring to a person's positive expectation about the future despite the current difficulties and setbacks (Carver and Scheier, 2002). More specifically, it is a personal proclivity to believe that one will normally experience good events in life and escape bad outcomes. In EFL contexts, optimism refers to a teacher's belief that he/she can improve students' learning by highlighting academics and learning, trusting parents, and students to get involved in the process, and believing in his/her capacity to defeat problems and resiliently react to failures (Hoy et al., 2008). Optimistic EFL teachers stay strong and determined in the face of challenges and adversities and envision positive and desirable outcomes for their actions (Pathak and Lata, 2018). Consequently, they do their best to execute instruction perfectly ignoring the setbacks.

### The Dimensions of Optimism

**Figure 1:**
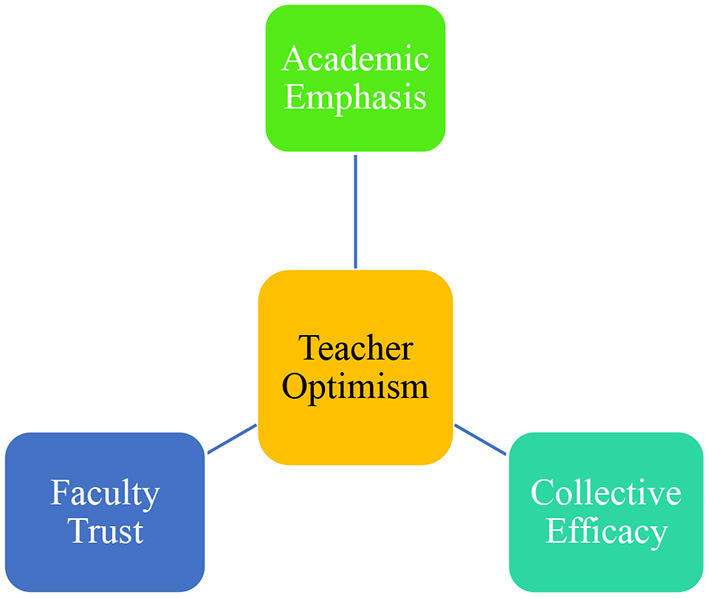
The dimensions of teacher optimism (Hoy et al., 2006).

Three dimensions have been proposed for teachers' optimism including *academic emphasis, faculty trust*, and *collective efficacy* (Figure 1). Academic emphasis concerns teachers' enacted behavior inspired by their beliefs in generating learning and academic success in students *via* an optimistic classroom culture. Faculty trust refers to teachers' confidence in students and parents to get engaged in the learning process. This involvement leads to establishing high academic standards for learning endorsed and favored by both students and parents. As the last dimension, collective efficacy refers to teachers' belief in their ability to execute instruction efficiently and cause student achievement (Hoy et al., 2006; Hoy and Miskel, 2013). It is essential to note these dimensions are in a reciprocal and interactional relationship each influencing and shaping the other two.

### Teacher Commitment

Teacher commitment is a mental association that a person creates between his/her beliefs and work in a way that the whole faithfulness is directed toward his/her profession (Lu, 2021). It is a pivotal element of successful teaching as it helps in establishing a learning environment where the students' achievement level and performance are improved significantly (Altun, 2017). As put by Sarikaya and Erdogan (2016), commitment manifests itself when a teacher feels and has a full obligation to take extra duties and tasks in his/her work so that positive outcomes are accomplished. Hence, it can be argued that teacher commitment in L2 education refers to a teacher's capability to accept a school's objectives with passion and energy, mentally identifies him/herself with the job, takes operational steps to improve instruction, and forms an emotional connection to the profession. It encompasses a sense of obligation to the school, students, vocation maintenance, proficient knowledge base, and teaching career (Crosswell and Elliott, 2004; Han, 2021b). As a fundamental core and a well-spring of instructional excellence/quality, teacher commitment improves students' achievement and participation, teachers' work performance, and school's quality. So, it is critical to establish, maintain, and promote this psycho-emotional variable in EFL teachers worldwide.

### Features of a Committed Teacher

There exists a number of characteristics for committed teachers. They basically make the constant attempt and have a desire for greatness, have strong interpersonal communication abilities, care about instruction progress, learning, and achievement (Altun, 2017). Moreover, committed teachers incline to consider students' needs and wants, know how to motivate students, can urge learners to participate in class activities, tend to fulfill school's short-term and long-term goals (Wang and Guan, 2020; Lu, 2021). Last but not the least, they are dedicated to faculty and its improvement, establish a positive learning climate, are mentally connected to their work, and are passionate about taking extra steps to promote teaching and learning cycle (Sarikaya and Erdogan, 2016).

### Work Engagement: Definitions and Dimensions

The concept of work engagement is grounded in work engagement theory that highlights the importance of personal enjoyment, vitality, and enthusiasm in work which drive the person forward in his/her job performance (Schaufeli et al., 2009; Han and Wang, 2021).

**Figure 2:**
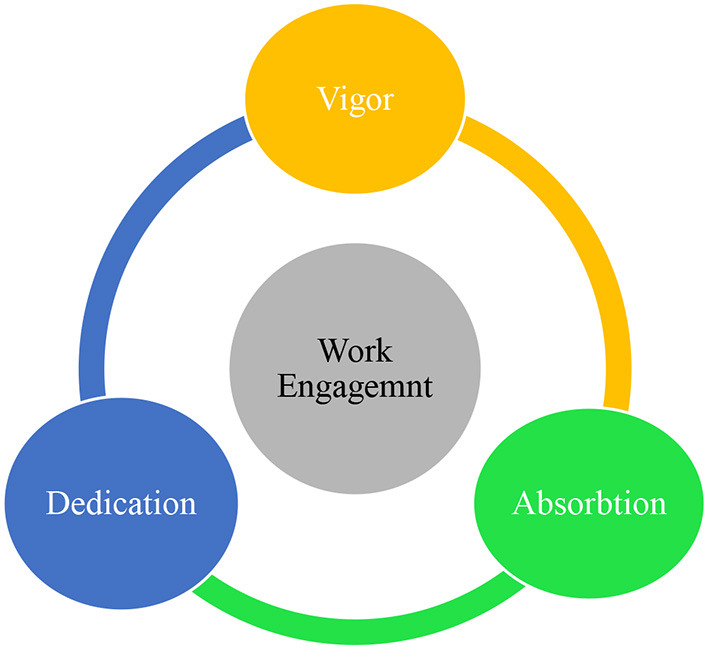
The dimensions of work engagement (Schaufeli et al., 2002).

It is a mental state pertaining to work that encompasses vigor, dedication and absorption (Schaufeli et al., 2009; Wang and Guan, 2020). It is the counterpart of burnout in that while burnout has harmful impacts on one's work performance, work engagement is a positive side of work that positively influences the person and the organization (González-Romá et al., 2006). In simple words, work engagement concerns how an individual dedicates time and energy to accomplish a task that is influenced by many internal and external factors (Han and Wang, 2021). According to Field and Buitendach (2012), teachers' work engagement is a positive emotional state inside the person that reflects their work life, performance, gratification, and quality. As pinpointed by Schaufeli et al. (2002), the construct has three dimensions of ***vigor*** which concerns having the willingness, energy, resilience, persistence in work, ***dedication*** that is a sense of work inspiration, enthusiasm, importance, pride, and challenge, and ***absorption*** which refers to being deeply involved in the work in such a way that one vehemently relishes working relentlessly as time passes (Figure 2). Furthermore, it is worth noting that the notion of work engagement is different from workaholism in the sense that engagement is a positive feature that generates positive outcomes, whereas workaholism is a negative trait does that causes harm and leads to job burnout. Based on these dimensions, it can be claimed that teachers' level of optimism and commitment is strongly related to and influences the quality and extent of work engagement in L2 teaching which has largely been ignored in the literature.

### Correlates of Teacher Work Engagement

In the available literature of work engagement in language education, the construct has been studied and associated with a number of factors and variables including burnout, self-efficacy, reflection, emotion-regulation strategies, psychological well-being, positive emotion, social support, resilience, job resources, and identity (e.g., Minghui et al., 2018; Shirazizadeh and Karimpour, 2019; Van Der Want et al., 2019; Greenier et al., 2021; Han and Wang, 2021). It has been shown that teachers' inner emotions like being optimistic and confident of their teaching abilities will influence their engagement level at work (Minghui et al., 2018). Likewise, research shows that teachers' optimism and commitment can reflect themselves in their occupational performance, satisfaction, problem-solving, and engaggenent (Field and Buitendach, 2012; San and Tok, 2017; Kristiana et al., 2018; Han, 2021a). Other correlates have been approved in the previous studies mentioned earlier in this review article. Yet, there are still many other related variables such as agency, willingness to communicate (WTC), and interpersonal communication skills which need empirical investigations to make their linkage to work engagement more vivid.

### Implications, Research Gaps, and Future Directions

In this mini-review study, it was argued that EFL teachers' optimism and commitment are directly and strongly associated to their quality and degree of work engagement. It was also maintained that each of these constructs has different dimensions influenced by numerous internal and external factors in L2 education. This has led to the conduct of various studies on each of the variables and their contribution to successful instruction and learning. As a result, the present review can offer a number of practical implications for EFL teachers, teacher educators, program designers, school principals, and L2 researchers. The study can be beneficial for EFL teachers in that it can raise their awareness and understanding of positive emotions and their role in teaching and learning processes. Moreover, EFL teachers will realize that their optimism and commitment to teaching can make a meaningful difference in their teaching quality as well as students' learning. Likewise, teacher trainers can use the ideas in this review and devise training courses for novice and experienced EFL teachers and teach different strategies to improve and boost EFL teachers' positive emotions, especially their optimism and commitment to teaching aside from pedagogical and methodological concerns. Additionally, program designers can offer workshops, seminars, and webinars to EFL teachers in which various ways of being optimistic and committed to instruction are taught professionally. School principals as important agents in education can also use the propositions made in this study and assist EFL teachers in being optimistic, committed, and engaged in work *via* providing a positive, democratic setting which has all the required facilities for teachers to execute instruction efficiently. Finally, L2 researchers can take advantage of this review article in that they can run replicate and complementary research focusing on the existing gaps in this line of inquiry. As stated, the majority of the studies on optimism, commitment, and work engagement are correlational and one-shot. Hence, future scholars can conduct qualitative and mixed-methods studies to make the picture more comprehensive. The role of cultural factors has also been ignored in this line of research, so future studies are recommended to explore the effect of culture on these three constructs. Furthermore, the developmental trajectories of each of these variables can be studied using portfolio, diary, and journal writing by EFL teachers of various experiences. In a similar manner, the possible impact of teachers' interpersonal communication skills such as stroke, credibility, clarity, care, confirmation and the like on EFL teachers' degree of optimism, commitment, and work engagement can be interesting avenues for research. Finally, the relationship between teachers' identity, agency, demographic factors, and the three variables presented in this study can also be a direction for future research. These ideas show that working on these three variables is still fresh in EFL contexts and more attention called for by L2 researchers all around the globe.

## Author Contributions

YD conceptualized and drafted the initial manuscript. YK and JX proofread and approved the final version to submit to Frontiers in Psychology. Both authors listed have made a substantial, direct, and intellectual contribution to the work and approved it for publication.

## Funding

This study was funded by FLTRP Project on Foreign Languages Teaching and Research entitled Edusemiotic Terms and Translations (Grant no. HXKT 20200017) of China and also supported by the University-level Scientific Research Project entitled Research on the Univerisity Students' Wellbeing of Anhui Province during the Pandemic Crisis (Grant no. XWY-202134) of Chaohu University, China.

## Conflict of Interest

The authors declare that the research was conducted in the absence of any commercial or financial relationships that could be construed as a potential conflict of interest.

## Publisher's Note

All claims expressed in this article are solely those of the authors and do not necessarily represent those of their affiliated organizations, or those of the publisher, the editors and the reviewers. Any product that may be evaluated in this article, or claim that may be made by its manufacturer, is not guaranteed or endorsed by the publisher.

## References

[B1] Altun M.. (2017). The effects of teacher commitment on student achievement: a case study in Iraq. Int. J. Acad. Res. Bus. Social Sci. 7, 417–426. 10.6007/IJARBSS/v7-i11/34753475

[B2] Asgari A. Rahimi S. (2014). Teachers' academic optimism: confirming a new construct. Int. J. Sci. Manag. Dev. 2, 105–109.

[B3] Benevene P. De Stasio S. Fiorilli C. (2020). Well-being of school teachers in their work environment. Front. Psychol. 11:1239. 10.3389/fpsyg.2020.01239 32903655 PMC7438736

[B4] Burić I. Macuka I. (2018). Self-efficacy, emotions and work engagement among teachers: a two wave cross-lagged analysis. J. Hap. Stud. 19, 1917–1933. 10.1007/s10902-017-9903-9

[B5] Carver C. Scheier M. (2002). “Optimism,” in Handbook of Positive Psychology, eds C. R. Snyder, and S. J. Lopez (New York, NY: Oxford University Press), 231–256.

[B6] Crosswell L. Elliott R. (2004). “Committed teachers, passionate teachers: the dimension of passion associated with teacher commitment and engagement,” in AARE Conference 2004 (Melbourne, VIC), 1–12.

[B7] Field L. K. Buitendach J. H. (2012). Work engagement, organizational commitment, job resources and job demands of teachers working within disadvantaged high schools in Kwazulu-Natal, South Africa. J. Psychol. Afr. 22, 87–95. 10.1080/14330237.2012.10874525

[B8] González-Romá V. Schaufeli W. B. Bakker A. B. Lloret S. (2006). Burnout and work engagement: Independent factors or opposite poles? J. Voc. Behav. 68, 165–174. 10.1016/j.jvb.2005.01.003

[B9] Greenier V. Derakhshan A. Fathi J. (2021). Emotion regulation and psychological well-being in teacher work engagement: a case of British and Iranian English language teachers. System 97:102446. 10.1016/j.system.2020.102446

[B10] Han K.. (2021a). Fostering students' autonomy and engagement in EFL classroom through proximal classroom factors: autonomy-supportive behaviors and student-teacher relationships. Front. Psychol. 12:767079. 10.3389/fpsyg.2021.767079 34744946 PMC8564368

[B11] Han K.. (2021b). Students' well-being: the mediating roles of grit and school connectedness. Front. Psychol. 12:787861. 10.3389/fpsyg.2021.787861 34867697 PMC8637869

[B12] Han Y. Wang Y. (2021). Investigating the correlation among Chinese EFL teachers' self-efficacy, work engagement, and reflection. Front. Psychol. 12:763234. 10.3389/fpsyg.2021.763234 34803845 PMC8603392

[B13] Hoy A. W. Hoy W. K. Kurz N. M. (2008). Teacher's academic optimism: the development and test of a new construct. Teach. Teach. Educ. 24, 821–835. 10.1016/j.tate.2007.08.004

[B14] Hoy W. K. Miskel C. G. (2013). Educational Administration: Theory, Research and Practice. New York, NY: McGraw Hill.

[B15] Hoy W. K. Tarter C. J. (2011). Positive psychology and educational administration: An optimistic research agenda. Educ. Admin. Q. 47, 427–447. 10.1177/0013161X10396930

[B16] Hoy W. K. Tarter C. J. Hoy A. W. (2006). Academic optimism of schools: a force for student achievement. Am. Educ. Res. J. 43, 425–446 10.3102/00028312043003425

[B17] Huang X. Lee J. C. K. Zhang Z. Wang J. (2016). Teacher Commitment in Northwest China. Rotterdam: Sense Publishers. 10.1007/978-94-6300-232-5_14

[B18] Kristiana I. F. Ardi R. Hendriani W. (2018). “What's behind the work engagement in teaching practice,” in Proceedings of the 3rd International Conference on Psychology in Health, Educational, Social, and Organizational Settings (ICP-HESOS 2018) - Improving Mental Health and Harmony in Global Community, 267–275. 10.5220/0008588102670275

[B19] Lu D.. (2021). EFL teachers' optimism and commitment and their contribution to students' academic success. Front. Psychol. 12:752759. 10.3389/fpsyg.2021.752759 34733218 PMC8558303

[B20] MacIntyre P. D. Gregersen T. Mercer S. (2019). Setting an agenda for positive psychology in SLA: theory, practice, and research. Modern Lang. J. 103, 262–274. 10.1111/modl.12544

[B21] McIntyre T. McIntyre S. Francis D. (2017). Educator Stress. New York, NY: Springer. 10.1007/978-3-319-53053-6

[B22] Minghui L. Lei H. Xiaomeng C. Potměšilc M. (2018). Teacher efficacy, work engagement, and social support among Chinese special education school teachers. Front. Psychol. 9:648. 10.3389/fpsyg.2018.00648 29867634 PMC5949842

[B23] Pajares F.. (2001). Toward a positive psychology of academic motivation. J. Educ. Res. 95, 27–35. 10.1080/00220670109598780

[B24] Pathak R. Lata S. (2018). Optimism in relation to resilience and perceived stress. J. Psychosoc. Res. 13, 359–367. 10.32381/JPR.2018.13.02.10

[B25] Safari M. Soleimani N. (2019). The relationship between teachers' academic optimism culture and student achievement in junior high schools in Tehran. Biannual J. Educ. Exp. 1, 43–58.

[B26] San B. Ç. Tok T. N. (2017). The relationship between teachers'work engagement and organizational commitment. Pamukkale Univ. J. Social Sci. Inst. Ocak 26, 355–370. 10.5505/pausbed.2017.37232

[B27] Sarikaya N. Erdogan Ç. (2016). Relationship between the instructional leadership behaviors of high school principals and teachers' organizational commitment. J. Educ. Pract. 7, 72–82.

[B28] Schaufeli W. B. Bakker A. B. Van Rhenen W. (2009). How changes in job demands and resources predict burnout, work engagement and sickness absenteeism. J. Org. Behav. 30, 893–917. 10.1002/job.59525855820

[B29] Schaufeli W. B. Salanova M. González-Rom,á V. Bakker A. (2002). The measurement of engagement and burnout: a two sample confirmatory factor analytic approach. J. Hap. Stud. 3, 71–92. 10.1023/A:1015630930326

[B30] Seligman M.. (2006). Learned Optimism: How to Change Your Mind and Your Life. New York, NY: Pocket Books.

[B31] Seligman M. E. P.. (2011). Flourish: A Visionary New Understanding of Happiness and Well-Being. New York, NY: Free Press.

[B32] Sezgin F. Erdogan O. (2015). Academic optimism, hope and zest for work as predictors of teacher self-efficacy and perceived success. Educ. Sci. 15, 7–19. 10.12738/ESTP.2015.1.2338

[B33] Shirazizadeh M. Karimpour M. (2019). An investigation of the relationships among EFL teachers' perfectionism, reflection and burnout. Cogent Educ. 6:1667708. 10.1080/2331186X.2019.1667708

[B34] Smith P. A. Hoy W. K. (2007). Academic optimism and student achievement in urban elementary schools. J. Educ. Admin. 45, 556–568. 10.1108/09578230710778196

[B35] Van Der Want A. C. Den Brok P. Beijaard D. Brekelmans M. Claessens L. C. Pennings H. J. (2019). The relation between teachers' interpersonal role identity and their self-efficacy, burnout and work engagement. Prof. Dev. Educ. 45, 488–504. 10.1080/19415257.2018.1511453

[B36] Wang Y. L. Derakhshan A. Zhang L. J. (2021). Researching and practicing positive psychology in second/foreign language learning and teaching: the past, current status and future directions. Front. Psychol. 12, 1–10. 10.3389/fpsyg.2021.731721 34489835 PMC8417049

[B37] Wang Y. L. Guan H. F. (2020). Exploring demotivation factors of Chinese learners of English as a foreign language based on positive psychology. Rev. Argent. Clin. Psicol. 29, 851–861. 10.24205/03276716.2020.116

